# Accelerated diversification is related to life history and locomotion in a hyperdiverse lineage of microbial eukaryotes (Diatoms, Bacillariophyta)

**DOI:** 10.1111/nph.15137

**Published:** 2018-04-06

**Authors:** Teofil Nakov, Jeremy M. Beaulieu, Andrew J. Alverson

**Affiliations:** ^1^ University of Arkansas 1 University of Arkansas, SCEN 601 Fayetteville AR 72701‐1201 USA

**Keywords:** anisogamy, diatoms, diversification, life history, motility, oogamy

## Abstract

- Patterns of species richness are commonly linked to life history strategies. In diatoms, an exceptionally diverse lineage of photosynthetic heterokonts important for global photosynthesis and burial of atmospheric carbon, lineages with different locomotory and reproductive traits differ dramatically in species richness, but any potential association between life history strategy and diversification has not been tested in a phylogenetic framework.
- We constructed a time‐calibrated, 11‐gene, 1151‐taxon phylogeny of diatoms – the most inclusive diatom species tree to date. We used this phylogeny, together with a comprehensive inventory of first–last occurrences of Cenozoic fossil diatoms, to estimate ranges of expected species richness, diversification and its variation through time and across lineages.
- Diversification rates varied with life history traits. Although anisogamous lineages diversified faster than oogamous ones, this increase was restricted to a nested clade with active motility in the vegetative cells.
- We propose that the evolution of motility in vegetative cells, following an earlier transition from oogamy to anisogamy, facilitated outcrossing and improved utilization of habitat complexity, ultimately leading to enhanced opportunity for adaptive divergence across a variety of novel habitats. Together, these contributed to a species radiation that gave rise to the majority of present‐day diatom diversity.

## Introduction

Patterns of species richness across time, space and clades are commonly linked to evolutionary innovations and ecological opportunities that are thought to influence the rates of speciation and extinction. Changes in life history can affect developmental or reproductive strategies and have consequences for reproductive success and fitness, whereas traits related to locomotion—which are tightly linked to life history in many taxa—can improve dispersal abilities and facilitate range expansions, range shifts or colonizations of previously unavailable habitats. As a result, transitions in life history (e.g. selfing or outcrossing), locomotion (e.g. sedentary or mobile) or their interactions are often correlated with shifts in species diversification (e.g. Leclère *et al*., 2009; Goldberg *et al*., 2010; Ikeda *et al*., 2012). Numerous life history traits have been linked to net diversification (birth − death, *r *= *b* − *d*) in vascular plants. These include features associated with longevity (annual vs perennial, Drummond *et al*., 2012), seed dispersal (Leslie *et al*., 2013; Beaulieu & O'Meara, 2016) and mechanisms that promote outcrossing, such as self‐incompatibility (Goldberg *et al*., 2010) and heterostyly (de Vos *et al*., 2014). Similar patterns are also evident in animals. In insects, for example, the evolution of complete metamorphosis (Rainford *et al*., 2014), as well as its subsequent reduction in some groups (Cieslak *et al*., 2014), have been associated with increases in net diversification (but see Condamine *et al*., 2016). In hydrozoans, two alternative life cycles, one with and one without a mobile life history stage, have been maintained by species selection related to lower extinction rates in lineages with a medusa or medusoid free‐swimming stage that facilitates dispersal (Leclère *et al*., 2009). Owing to its role in dispersal, mating and interactions with environmental conditions in general, locomotion has also been linked with diversification. By facilitating long‐range dispersal, the presence and properties of motile life history stages can increase gene flow across populations and, in turn, reduce rates of speciation (e.g. Palumbi, 1992, 1994 and references therein). On the other hand, limited locomotory abilities could restrict gene flow between divergent and potentially locally adapted populations, thereby increasing rates of speciation (Duda & Rolán, 2005; Ikeda *et al*., 2012). Such interactions among life history, motility and ecology are common across the tree of life and are especially important in microbial eukaryotes (protists) in which the ability of self‐propelled locomotion often changes with alternating life history stages (Hoek *et al*., 1995).

One such interaction has played out in the evolutionary history of diatoms (Bacillariophyta), an ecologically, functionally and morphologically diverse clade of stramenopile algae (Andersen, 2004). Diatoms are thought to be ancestrally planktonic and oogamous, with nonmotile vegetative cells suspended in a dilute environment, necessitating motile flagellated gametes to reproduce successfully (Drebes, 1977; Round *et al*., 1990; Chepurnov *et al*., 2004). However, flagellated male gametes were lost at least twice: once in the common ancestor of the pennate diatoms – a large clade of predominantly benthic species whose gametes are behaviorally dimorphic and move via pseudopodia (Davidovich *et al*., 2010; Sato *et al*., 2011; Kaczmarska *et al*., 2017), and once in *Ardissonea crystallina* – a member of a benthic marine clade referred to as toxariids (Davidovich *et al*., 2017). Active, directed motility in *vegetative* cells evolved subsequently in a clade nested within pennate diatoms (raphid pennate diatoms; Harper, 1977; Round *et al*., 1990). Motility of these cells is enabled by a longitudinal slit through the cell wall called a *raphe,* which is lined with actin–myosin protein complexes (Poulsen *et al*., 1999) that move the cell across a substrate by displacing strands of extracellular mucilaginous secretions (Harper, 1977; Round *et al*., 1990). Diatoms outside this lineage can use these secretions for attachment and, in some cases, movement (Pickett‐Heaps *et al*., 1986, 1991; Kooistra *et al*., 2003), but the range, velocity and responsiveness of cells with raphe‐enabled motility are unmatched by other types of motility found in nonraphid diatoms (Consalvey *et al*., 2004). In all, three combinations of mode of sexual reproduction and locomotion of vegetative cells are known in diatoms: oogamous and nonmotile lineages (the paraphyletic group of ‘centric’ diatoms, excluding anisogamous toxariids), anisogamous and nonmotile lineages (the paraphyletic group of ‘araphid’ pennate diatoms, including the anisogamous toxariids), and anisogamous and motile lineages (the clade of raphid pennate diatoms) (Round *et al*., 1990; Theriot *et al*., 2015; Parks *et al*., 2017).

Comparisons between diatom groups with different combinations of reproductive and locomotory traits reveal drastic differences in species richness, with anisogamous diatoms far outnumbering oogamous diatoms and, within the former, lineages with motile vegetative cells (specifically, the raphid pennates) far outnumbering lineages with nonmotile vegetative cells (Guiry & Guiry, 2017; Kociolek *et al*., 2017). This apparent disparity in species richness and rates of diversification was first investigated by James Small (e.g. Small, 1945a,1945b, 1950), whose work on the geological duration of fossil taxa revealed differences in species turnover between diatoms with radially vs bilaterally symmetrical cell walls (Small, 1950; see also Van Valen, 1973). Small's results were framed in terms of cell symmetry, the primary basis of diatom classification at the time, but the division between ‘centric’ and ‘pennate’ also largely mirrors the split between oogamous and anisogamous diatoms. An added layer of complexity comes from common *in vitro* observations of selfing (homothally) in centric diatoms and the prevalence of outcrossing (heterothally) in pennates (Chepurnov *et al*., 2004 and references therein). As a result, Small's early findings of differences in diversification dynamics between centric and pennate diatoms also apply to variation in the mode of sexual reproduction and outcrossing: oogamous diatoms (generally capable of selfing *in vitro*) diversified more slowly than anisogamous diatoms (generally incapable of selfing *in vitro*).

These considerations suggest that life history—specifically the mode of sexual reproduction—is one of the key factors driving the observed disparity in species richness across diatom groups. However, directed motility of vegetative cells within the anisogamous lineage (raphid diatoms) conferred many ecological advantages as well, including the ability to quickly respond to changes in light and nutrient availability, diurnal and tidal migrations, and pheromonal movements (Palmer & Round, 1967; Sato *et al*., 2011; Cohn *et al*., 2015; Bondoc *et al*., 2016a,2016b). The aggregated effect of these benefits suggests an alternative hypothesis—namely, that the primary driver of species richness in raphid pennate diatoms was active motility of vegetative cells, the evolution of which fundamentally altered both inter‐ and intraspecies interactions (Drebes, 1977; Harper, 1977; Kingston, 1999), enabled the colonization of novel habitats (Palmer & Round, 1967; Sims *et al*., 2006) and allowed them to better exploit habitat heterogeneity (Consalvey *et al*., 2004; Cohn *et al*., 2015). Such a scenario would imply that Small's discovery of faster diversification of anisogamous diatoms is a result of faster diversification in the nested lineage of actively motile raphid pennate diatoms, analogous to the existence of an important unconsidered (or ‘hidden’) trait (Beaulieu & O'Meara, 2016) – in this case, a novel means of locomotion within a larger clade with a derived mode of sexual reproduction.

We tested these hypotheses using a combination of phylogenetic and paleontological data, combining a large dataset of first–last occurrences of marine Cenozoic fossil diatoms with an 11‐gene phylogeny for 1151 diverse diatom taxa, which represents the most comprehensive analysis of the diatom phylogeny to date. We found that, although anisogamous diatoms diversified faster than oogamous diatoms, the higher rates were largely restricted to the nested clade of actively motile species. We propose that the evolution of directed motility enabled improved utilization of habitat complexity, colonization of novel habitats and more frequent or efficient sexual reproduction, ultimately resulting in increased genetic diversity, greater potential for evolutionary change and accelerated species diversification in the lineage of raphid pennate diatoms.

## Materials and Methods

### Phylogenetic dataset assembly

We compiled data for 11 genes that exhaust the set of markers with substantial representation in publicly available databases (Sorhannus & Fox, 2012; Theriot *et al*., 2015; Ruck *et al*., 2016). These included two nuclear rRNA genes (18S and 28S rRNA), seven plastid genes (16S rRNA, *atp*B, *psa*A, *psa*B, *psb*A, *psb*C and *rbc*L) and two mitochondrial genes (*cob* and *cox*I) (Supporting Information Table S1). The combined data from these genes encompasses as much diatom diversity as possible, whilst maximizing the power to resolve both old and recent divergences (Theriot *et al*., 2015). After removing environmental sequences, which are commonly identified to the genus level only, we used Usearch (Edgar, 2010) to identify and remove duplicate accessions (Table S1). We then aligned the sequences and performed several rounds of filtering to remove misidentified sequences, taxonomic duplicates and unnamed species that were identical (or nearly so) to a named sister taxon (based on zero or near‐zero branch lengths). Decisions regarding the removal of misidentified sequences were made on the basis of the manual inspection of maximum likelihood (ML) trees built with RAxML (Stamatakis, 2014). When an accession appeared correctly aligned, but was reconstructed phylogenetically far from its expected position (i.e. it fell outside the expected genus or family), we flagged it as misidentified and removed it from the alignment. These decisions were made in consultation with the primary literature to ensure that an accession in question had not been recently transferred to a different taxonomic group or had been previously known to have an unexpected phylogenetic placement compared with that suggested by its name. These checks were important because many diatom genera are not monophyletic and can comprise distant relatives that are taxonomically united on the basis of convergent (Alverson *et al*., 2007; Ruck & Theriot, 2011) or plesiomorphic (Ruck *et al*., 2016) characters. For taxonomic duplicates, we removed the accession with fewer genes, and for cases in which a pair of taxonomic duplicates had different gene complements, we combined their data into a single accession. Finally, we dropped all accessions represented by a single gene that was not 18S or *rbc*L, the two genes with the highest taxon occupancy in the dataset. Preliminary tree searches showed that these types of singletons were often inconsistently or erroneously placed phylogenetically. To enable the estimation of branch lengths at the split between diatoms and Parmales (Bolidophyceae), we included nuclear ribosomal RNA and plastid gene data for 13 additional outgroups from the stramenopile classes Chrysophyceae, Phaeophyceae, Xanthophyceae, Raphidophyceae, Eustigmatophyceae and Pelagophyceae. Data files, phylogenies and scripts are available from a Zenodo online repository: https://doi.org/10.5281/zenodo.583628.

### Multiple sequence alignment, model partitioning and phylogenetic inference

Ribosomal RNA genes (16S, 18S and 28S) were aligned on the basis of covariance models of secondary structure (Cannone *et al*., 2002; Nawrocki *et al*., 2009). For the nuclear 18S and plastid 16S genes, we used covariance models for Eukarya and Bacteria, respectively, and removed sequences shorter than 250 nucleotides in length. For the 28S gene fragment, we used a covariance model developed from aligned full‐length 28S sequences from stramenopiles (Nakov *et al*., 2014). We masked secondary structure alignments to remove sites with low probability of positional homology based on the covariance models (Nawrocki *et al*., 2009). Protein‐coding genes were aligned with Mafft v.6.864b (Katoh & Standley, 2013) and adjusted manually to resolve gaps caused by incomplete codons. Single‐gene alignments were then concatenated into a single 11‐gene alignment.

We split the 11‐gene alignment into seven partitions (one for the combined rRNA (*n *=* *1 partition), one per codon position for the combined plastid genes (*n *=* *3 partitions) and one per codon position for the combined mitochondrial genes (*n *=* *3 partitions) (Table S2)) and identified the best nucleotide substitution model using Iq‐tree v.1.4.1 (Nguyen *et al*., 2015). Tree searches were performed with Iq‐tree using the above partitioning scheme and substitution models and the edge‐proportional model to accommodate between‐partition differences in evolutionary rate. We performed 100 ML optimizations with default settings, except for the strength of perturbation of the nearest‐neighbor interchange (Iq‐tree option: ‐pers, default = 0.5), which we varied between 0.3 and 0.6 with 0.05‐unit increments. Support for the inferred relationships was assessed with 10^3^ UltraFast bootstrap replicates (Minh *et al*., 2013). The interpretation of the UltraFast bootstrap values differs from that of a standard nonparametric bootstrap resampling in that only nodes with UltraFast bootstrap support ≥95% are considered to be strongly supported.

### Time calibration

We time calibrated the phylograms using treePL (Sanderson, 2002; Smith & O'Meara, 2012) and 38 calibration points taken from first appearances of diatom lineages in the fossil record (Table S3). Most first‐appearance data came from the primary literature (Gersonde & Harwood, 1990; Harwood & Gersonde, 1990; Harwood & Nikolaev, 1995; Sims *et al*., 2006; Harwood *et al*., 2007), and many have been used previously (Medlin, 2015). However, given the size of our phylogeny and the importance of having calibrations distributed throughout the tree and covered time span, we also compiled first‐appearance records from the Neptune/Chronos database (Lazarus, 1994) which includes data from the Deep Sea Drilling Project (DSDP) and Ocean Drilling Project (ODP).

To construct calibration bounds, we followed a procedure that uses the difference between the first occurrences of two sister lineages (ghost lineage time), or the difference between the earliest and second earliest occurrence of a lineage (penultimate distance time) (Norris *et al*., 2015). For example, the first occurrences in the fossil record of the sister taxa *Aulacoseira* (*Archepyrgis*) and *Melosira* are 115 million yr ago (Ma) and 125 Ma, respectively (Table S3), resulting in a ghost lineage time of 10 million yr (Myr). Following Norris *et al*. (2015), a prior distribution for this node can be constructed using a lognormal distribution with a mean set to half the ghost lineage time (5 Myr in this case), a standard deviation of 1.814 Myr and an offset given by the earlier of the two first occurrences (*Melosira*, 125 Ma in this case). We used the 5% and 95% quantiles of this distribution as the minimum and maximum bounds for the age of the calibrated node in treePL. We used minimum age bounds only when there was not enough information to calculate the ghost lineage or penultimate distance time. We obtained a total of 21 calibrations with minimum and maximum bounds (including the most recent common ancestor (MRCA) of diatoms + Parmales and MRCA of all diatoms) and 17 calibrations with a minimum bound only. The majority of calibrations were within centric diatoms (19), with 11 in the araphid grade and six in the raphid clade.

To find the optimal rate‐smoothing parameter for the penalized likelihood time calibration (Sanderson, 2002), we used a random subsample and replace cross‐validation procedure (Smith & O'Meara, 2012) with a range of tested values for the smoothing parameter between 10^2^ and 10^−5^. Cross‐validation showed that a value of 10^−3^ provided the best smoothing, indicating substantial evolutionary rate variation across the phylogeny, as expected for a phylogenetic tree of this size and age. We used the calibrations described in Table S3 and the rate‐smoothing procedure described above to time calibrate the best tree and all bootstrap phylogenies for downstream analyses.

### Diversification rate at predefined clades

**Figure 1:**
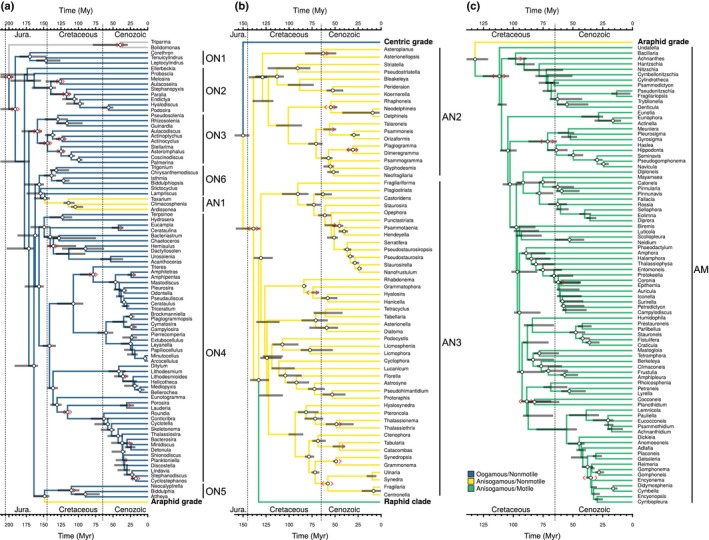
Time‐calibrated phylogeny of diatoms based on 11 genes with 1151 diatoms and six Parmales pruned down to one taxon per genus (*N *=* *234). The highest scoring tree, based on 100 maximum likelihood optimizations, is shown. In (a–c), branches are colored according to life history and motility. White circles at nodes indicate UltraFast bootstrap support ≥95%. Gray bars denote the 5% and 95% quantiles of the distribution of node ages across bootstrap replicates in millions of years (Myr). Red angular brackets denote calibrations, single ‘>’ for a minimum bound only and double ‘< >' for minimum and maximum bounds. Clade labels for oogamous nonmotile (ON), anisogamous nonmotile (AN) and anisogamous motile (AM) lineages match the groups in Figs 2(c) and 3(b–d).

Although the sample of 1151 taxa in our dataset encompasses a broad sample of diatom diversity and considerably expands upon previous efforts to reconstruct the diatom phylogeny, our species count still falls well short of the total estimated number of diatom species (see below). To accommodate unsampled diversity in our models, we pruned the phylogeny to include just one representative per genus (Fig. 1a–c), such that each tip was an unresolved polytomy representing the entire species diversity within that genus (*N *=* *234 genera; ‘genus‐level’ analysis). This strategy breaks down when genera are nonmonophyletic, and so we also performed analyses with genera grouped into more inclusive clades (*N *=* *45 clades; ‘clade‐level’ analysis).

We calculated net diversification rates using (1) the crown or stem age of clades across bootstrap trees, (2) the total number of species per lineage (see below), and (3) an estimate of the diatom‐wide relative extinction rate (or ‘extinction fraction’, ε *= d/b;* see below). We also calculated the 95% confidence interval for the expected size of a clade given its crown or stem age (Magallón & Sanderson, 2001).

The total number of diatom species is unknown, and recent approximations vary by orders of magnitude, from 2 × 10^4^ (Guiry, 2012) to 10^5^ (Mann & Vanormelingen, 2013), indicating that our phylogenetic hypothesis includes, at best, 5% of all diatom species. We therefore calculated the net diversification rate across a range of diversity estimates: (1) *N*_Total_ = 11 958 species, the AlgaeBase total number of taxonomically accepted names for all genera represented in the phylogeny (Guiry & Guiry, 2017); (2) *N*_Total_ = 10 569 species, the DiatomBase total number of taxonomically accepted names for extant species in all genera represented in the phylogeny (Kociolek *et al*., 2017); (3) *N*_Total_ = 20 000 species (Guiry, 2012); (4) *N*_Total_ = 30 000 species, the lower estimate of Mann & Vanormelingen (2013); and (5) *N*_Total_ = 100 000 species, the upper estimate of Mann & Vanormelingen (2013).

Standing diversity for analyses at the genus and clade levels was taken from both AlgaeBase and DiatomBase. We report results that used data from DiatomBase because it provides an estimate for the number of extant species, which is the quantity of interest for diversification models (Magallón & Sanderson, 2001). However, a comparison of extant diversity per genus from DiatomBase with total diversity per genus from AlgaeBase showed similar numbers, and both datasets provided qualitatively similar results.

Our calculations of net diversification required a fixed value for the relative extinction rate (Magallón & Sanderson, 2001). We followed the method of Foote (2000), as implemented in the R package paleotree v.2.6 (Bapst, 2012), to obtain an empirical estimate of relative extinction. This approach used the first and last occurrence of fossil species binned into discrete, nonoverlapping time intervals to calculate the instantaneous rates of speciation and extinction. Fossil data were taken from the Barron Diatom Catalog of first and last occurrences of Cenozoic diatoms (Lazarus *et al*., 2014). To obtain a single estimate of relative extinction for the entire set of fossils spanning the Cenozoic, we binned the data into discrete time intervals, calculated rates of speciation and extinction, and then averaged over time intervals. To assess the effect of interval duration on the estimates of relative extinction and downstream analyses, we repeated the calculations with bin durations of 0.5, 1.0, 2.5 and 5.0 Myr.

### Discrete shifts and temporal variation in diversification

To complement the analysis based on predefined taxonomic ranks, we used a rank‐free approach to identify discrete shifts in diversification rate that uses a stepwise Akaike information criterion (AIC) procedure (Medusa; Alfaro *et al*., 2009; Brown *et al*., 2016). The addition of a discrete shift to the Medusa diversification model increases the number of model parameters, and so more complex models were retained only when the improvement in AIC was greater than a threshold derived from the size of the phylogeny (*N *=* *234, ΔAICc = 6.7; ‘genus‐level’ analysis). To account for discordance between taxonomy and phylogeny, we repeated the analysis using a clade‐level phylogeny (*N *=* *45, ΔAICc = 2.3; ‘clade‐level’ analysis). We restricted the algorithm to test only birth–death models and allowed shifts in diversification at both stems and nodes of the phylogeny.

To summarize Medusa results and account for uncertainty in divergence times across bootstrap trees, we focused on breakpoints detected in ≥50% or ≥75% of bootstrap trees. We also calculated temporal trends of net diversification (*r* = birth − death), net turnover rate (τ = birth + death *sensu* Beaulieu & O'Meara (2016)) and relative extinction (ε = death/birth). We split the phylogenies into 1‐Myr intervals and obtained summary statistics for parameters of all branches intersecting an interval (Tank *et al*., 2015).

## Results

### Time‐calibrated phylogeny of diatoms

Consistent with previous multigene‐based (e.g. Theriot *et al*., 2015) and transcriptome‐based (Parks *et al*., 2017) phylogenies, we reconstructed the centric diatoms as a grade of five large clades (Figs 1a, S1). A clade of Corethrales + Leptocylindrales was sister to all other diatoms, and a clade of the multipolar diatoms *Attheya* and *Biddulphia* was sister to the clade of pennate diatoms (Figs 1a, S1). The diatom crown age was estimated at 190.4 Ma (mean across bootstrap trees), placing the origin of diatoms near the Triassic–Jurassic boundary (Fig. 1a). Origins of genus‐level diversity within the grade of centric diatoms largely pre‐dated the Cenozoic, with notable exceptions within Eupodiscales, Cymatosirales, Lithodesmiales and Thalassiosirales (Fig. 1a). These results suggested both temporal and lineage‐specific variation in the origination of genera, whereby genera within the radial centric grade diversified much earlier than their counterparts in the polar centric grade (Fig. 1a). The pennate and raphid lineages also had their origins in the Cretaceous, with genus originations distributed more evenly throughout the Cretaceous and Cenozoic (Fig. 1b,c).

### Accelerated diversification in diatoms with actively motile vegetative cells

**Figure 2:**
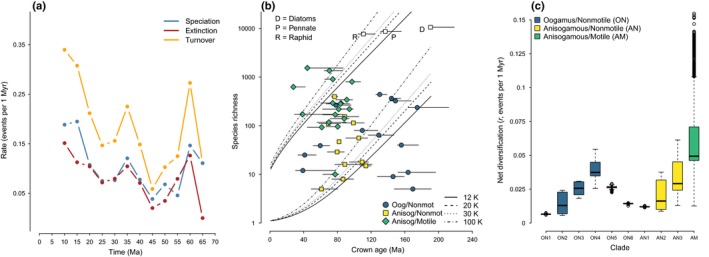
Diatom diversification based on fossil and phylogenetic data at predefined clades. (a) Rates of speciation (birth, *b*), extinction (death, *d*) and species turnover (τ *= b + d*) based on first–last occurrence data for Cenozoic diatoms from the Barron Diatom Catalog. (b) Confidence intervals (95%) for species richness of predefined diatom clades, based on crown ages, standing diversity and a relative extinction (ε = *d*/*b*) of ε = 0.751. Line types correspond to different approximations of total diatom diversity (see the Materials and Methods section). Species richness from DiatomBase is plotted against crown age (million years ago, Ma) and color coded by the combination of reproductive mode and locomotion. Error bars span the minimum and maximum crown ages of clades across bootstrap trees in million years (Myr). The clades of all diatoms (D), pennate diatoms (P) and raphid diatoms (R) are shown as open squares. Clades younger than 10 Myr or having fewer than five species are omitted. (c) Net diversification rates (*r = b–d*), averaged over bootstrap phylogenies and pooled based on the most inclusive monophyletic groups with a certain combination of reproductive and locomotory traits. Lines, boxes and whiskers represent medians, inter‐quartile range and quartile values + 1.5 × quartile values, respectively. See labels in Fig. 1 for contents of oogamous nonmotile (ON), anisogamous nonmotile (AN) and anisogamous motile (AM) groups. Oog, oogamous; Anisog, anisogamous; Nonmot, nonmotile. See Supporting Information Fig. S4 for an identical analysis with AlgaeBase instead of DiatomBase species richness.

The analysis of Cenozoic marine diatom fossils showed that both speciation (birth, *b*) and extinction (death, *d*) decreased during the first 20 Myr of the Cenozoic (Fig. 2a). The last 40–45 Myr were marked by a slow increase in turnover (*b *+ *d*) that first peaked at *c*. 35 Ma and then again towards the recent (Fig. 2a). These results were in agreement with recent paleontological studies based on similar data (Lazarus *et al*., 2014; Cermeño *et al*., 2015).

Using these data, we calculated that Cenozoic marine fossil diatoms diversified at a mean rate of *r *=* *0.029 (SD = 0.007) net speciation events per 1 Myr, with a relatively high mean relative extinction rate of ε = 0.751 (SD = 0.081; Table 1). These values were averages of calculations performed with the fossil data binned into discrete, nonoverlapping time intervals with durations of 0.5, 1.0, 2.5 and 5.0 Myr (Fig. S2). Comparison of results obtained with different interval durations indicated that the estimates of relative extinction varied with the granularity of the fossil data, being highest for 5‐Myr intervals and lowest for 0.5‐Myr intervals (Fig. S2). The most pronounced downstream effect of relative extinction calculated with different interval durations was noticed for young clades inferred to have diversified at a high rate (Fig. S3). For such clades, relative extinction calculated with a bin size of 5 Myr provided the most conservative values for net diversification (Fig. S3). Although this variation had a minor effect on downstream calculations of net diversification (Fig. S3), we nonetheless performed all downstream analyses with relative extinction averaged over the estimates obtained from the four different bin sizes (Table 1; Fig. S2).

**Table 1:** Instantaneous rates of speciation (birth, *b*) and extinction (death, *d*) based on first–last occurrence data of Cenozoic marine diatoms summarized over time

	Median	Mean	SD
Speciation (*b*)	0.093	0.097	0.009
Extinction (*d*)	0.086	0.085	0.005
Net diversification (*r = b − d*)	0.029	0.027	0.007
Relative extinction (ε *= d/b*)	0.731	0.751	0.081
Net turnover (τ *= d + b*)	0.193	0.190	0.030

The fossil data were binned into discrete, nonoverlapping intervals with durations of 0.5, 1.0, 2.5 and 5.0 million yr (Myr). Summary statistics were calculated for each granularity, and the reported values are averages of the median parameter estimates across bin sizes. See Supporting Information Fig. S2 for more details on parameter estimates at different granularities.

Using the above estimate of relative extinction averaged through time and across bin sizes (ε = 0.751), the inferred crown age of diatoms (mean = 190.4 Ma; Fig. 1) and different approximations of total diatom diversity, we calculated a minimum net diversification of *r *=* *0.044 events per 1 Myr per lineage (SD = 0.00033) for *N*_Total_ = 20 000 species and a maximum of *r *=* *0.052 (SD = 0.00039) for *N*_Total_ = 100 000 species. The 95% confidence limits on expected species richness under the least conservative scenario (*N*_Total_ = 100 000 species) indicated that the expected diversity of a clade with a crown age of 50 Myr could range from two to 218 species.

**Figure 3:**
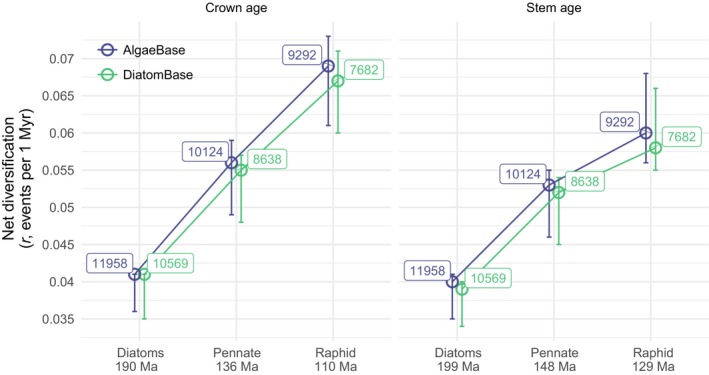
Net diversification (birth − death, *r = b − d*, events per million years) estimated from known standing diversity and crown or stem ages for all diatoms (ON + AN + AM), the bilaterally symmetrical clade (pennate diatoms, AN + AM) and the actively motile clade (raphid diatoms, AM). Shown are the mean values (circles) and minimum–maximum range (error bars) across bootstrap phylogenies. All calculations used relative extinction ε = 0.751 estimated from Cenozoic fossil data (Table 1). Numbers next to the points indicate the total number of species in the respective databases. Numbers below the clade names are the estimates of crown and stem ages in million years ago (Ma, Fig. 1). ON, oogamous nonmotile; AN, anisogamous nonmotile; AM, anisogamous motile.

Although the numbers of oogamous/nonmotile genera (ON; number of genera *N*_G_ = 86), anisogamous/nonmotile genera (AN; *N*_G_ = 61) and anisogamous/motile genera (AM, raphid; *N*_G_ = 85) included in our analyses were comparable (Fig. 1), at the species level, the number of described AM species (DiatomBase number of species, *N*_S_ = 9533) far exceeds the numbers of described ON (*N*_S_ = 1871) and AN (*N*_S_ = 759) diatoms (Figs 2b, 3). This unbalanced distribution of species‐level diversity was evident in the diversity‐by‐age plot (Fig. 2b). The raphid pennate (AM) lineage as a whole was more diverse than expected, or, at least, at the upper bound of expected diversity for *N*_Total_ = 100 000 species (Fig. 2b). The broader pennate diatom lineage (AN + AM) was also more diverse than expected when the assumed total diatom diversity was ≤ 20 000 species (Fig. 2b).

At a lower phylogenetic scale, only lineages within the actively motile raphid pennate (AM) clade had higher than expected species richness, whereas the species richness of clades within the grades of AN and ON diatoms either was within or below the bounds of expected species richness (Fig. 2b). The estimated diversification rate of raphid (AM) diatoms as a whole was 1.66 times greater than that of all diatoms (Fig. 3), and the diversification rate of AM diatoms was significantly higher than that of any of the AN and ON lineages, with rate ratios ranging between 1.6 and 10.7 (Tukey *post hoc* tests, *P*_adj_ < 0.001, Fig. 2c). These comparisons were obtained by pooling and averaging diversification rate estimates of clades grouped into the most inclusive monophyletic groups with a particular combination of life history and locomotory traits. For example, for the AM lineages that together form a single monophyletic group (raphid pennate diatoms), we averaged over all AM clades, whereas, as the AN and ON lineages are paraphyletic, we pooled clade‐level parameters into the most inclusive monophyletic groups (e.g. Corethrales + Leptocylindrales; Fig. 1). Results using stem rather than crown clade ages (Fig. 3) and alternative sources for the number of species, i.e. AlgaeBase instead of DiatomBase, agreed with these results (Figs 3, S4).

### Discrete shifts and temporal trends in diatom diversification

**Figure 4:**
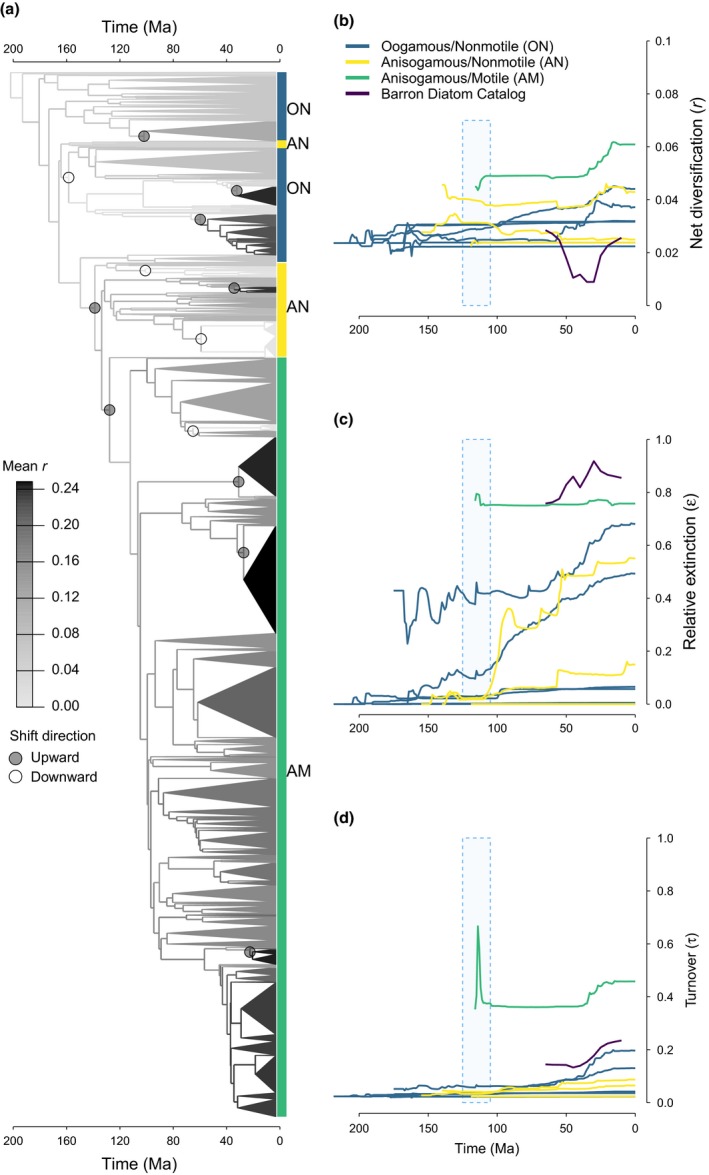
Discrete shifts and temporal trends of diversification across diatoms. (a) Medusa reconstruction of rates of (birth − death, *r = b − d*, events per million years) across the genus‐level phylogeny of diatoms + Parmales. Triangle size is proportional to the number of species per genus (from DiatomBase), and the shading corresponds to the estimated net diversification rate. Rate shifts detected in ≥50% of trees are shown. (b–d) Temporal trends in net diversification, relative extinction (ε *= d/b*) and turnover (τ = *b + d*) as estimated by the genus‐level Medusa analysis. Trend lines were calculated by slicing the bootstrap phylogenies into 1 million yr (Myr) intervals and averaging the branch‐associated parameters for each slice across trees. Trendlines are shown for the most inclusive clades with a combination of reproductive and locomotory traits, and match the groups in Fig. 2(c) and the clade labels in Fig. 1. Rates estimated from the Cenozoic marine fossil record (bin size, 5 Myr) were smoothed using nonparametric local polynomial regression (LOESS), but are otherwise not different from Fig. 2(a). The timing of the evolution of active motility (the range of crown age estimates for raphid pennates across bootstrap phylogenies) is shown as a blue rectangle. See Supporting Information Fig. S5 for an identical analysis with AlgaeBase instead of DiatomBase species richness. Ma, million years ago.

In the genus‐level analysis with DiatomBase species richness data, Medusa identified 23 rate shifts that occurred in at least 5% of the bootstrap trees. Of these, 13 were present in ≥50% and 10 were detected in ≥75% of trees (Fig. 4a). Most shifts occurred within the past *c*. 70 Myr, and upward shifts tended to be more recent, consistent with the Cenozoic increase in diatom diversity as a whole (Rabosky & Sorhannus, 2009; Lazarus *et al*., 2014; Cermeño *et al*., 2015), as well as many lineages of both ON (Alverson, 2014) and AM (Edwards, 1991) diatoms (Fig. 4a). Breakpoints at deeper internal branches were detected in Thalassiosirales (ON, upward shift), deeper within a clade of multipolar diatoms (ON, downward shift) and in two clades of AN diatoms (downward shifts, Fig. 4a). Two phylogenetically deep upward shifts coincided with the transitions from oogamy to anisogamy in the ancestor of pennate diatoms (frequency = 0.70, magnitude = 0.007) and with the evolution of active motility in the ancestor of raphid pennate diatoms (frequency = 0.69, magnitude = 0.014, Fig. 4a).

Averaged branch‐associated parameters binned into 1‐Myr intervals showed increases in the rates of net diversification, relative extinction and species turnover after the emergence of actively motile raphid pennate diatoms (Fig. 4b–d). Temporal trends in lineages with different life history strategies reinforced the finding of faster diversification in actively motile raphid diatoms (AM) compared with their nonmotile counterparts (ON and AN, Fig. 4b). This difference in diversification, however, was not caused by higher rates of speciation among raphid diatoms against a background of similar extinction rates, or the reverse, lower rates of extinction with comparable speciation. The difference instead was a result of increases in both the birth of new lineages and their extinction (turnover = birth + death, Fig. 4d). Accordingly, a higher fraction of species per unit time went extinct among AM lineages than in the ON and AN grades (Fig. 4c). We found qualitatively similar results when using species richness data from AlgaeBase instead of DiatomBase, and when we collapsed the phylogeny down to the 45 predefined clades used in the previous analysis (Figs 3, S4, S5).

## Discussion

Diversity patterns are frequently associated with evolutionary innovations and ecological opportunities that are thought to alter the rates at which species are born and lost through time. Transitions in locomotory and life history strategies are important traits that may underlie major shifts in diversification (Leclère *et al*., 2009; Goldberg *et al*., 2010; Ikeda *et al*., 2012; Cieslak *et al*., 2014; de Vos *et al*., 2014). Changes in life history may also drive shifts in locomotion (or vice versa) to optimally match these traits, which are often linked in microbial eukaryotes (Hoek *et al*., 1995).

Our results suggest that an interaction between motility and life history during diatom evolution may have promoted an increase in the rate of diversification of raphid pennate diatoms, a lineage that evolved a novel mechanism of directed motility in vegetative cells following the earlier loss of flagellated male gametes (Figs 2, 3, 4). At least 137 Ma (Fig. 1), the MRCA of pennate diatoms experienced a reduction in the morphological and behavioral differences between gametes, such that the typical egg‐and‐sperm oogamy present in the diatom ancestor, and largely preserved across the grade of ON (‘centric’) diatoms, was replaced with anisogamy. It is unclear what, if any, selective pressures led to the loss of the ancestral oogamous reproductive system. Oogamy is thought to be advantageous because a species can maximize the number of gametes and, as a result, successful copulation encounters by producing many small and highly motile male gametes, as well as zygote survivorship by producing one or a few large (and immobile) female gametes (Parker *et al*., 1972; Maynard Smith, 1978; Matsuda & Abrams, 1999; Bulmer & Parker, 2002; Togashi *et al*., 2012). In pennate diatoms, however, the flagellum of male gametes was lost, and dimorphism between gametes diminished, such that gametes in a copulation pair converged towards a similar, intermediate size (reviewed in Kaczmarska *et al*., 2013), but differed in their behavior (Drebes, 1977), with male gametes that moved by pseudopodia to search for a nonmotile female gamete (Davidovich *et al*., 2010, 2017; Sato *et al*., 2011; Kaczmarska *et al*., 2017). The evolution of the raphe and active motility in vegetative cells enabled gametangia, rather than gametes, to search for mating partners, promoting further reduction in sexual dimorphism and gamete mobility. An added benefit of raphe‐enabled locomotion is linked to the evolution of a system resembling internal fertilization, whereby copulation occurs between closely spaced cells protected by a mucilaginous ‘copulation envelope’ (Drebes, 1977; Round *et al*., 1990; Kaczmarska *et al*., 2013). Thus, in raphid pennate diatoms, the choice of a compatible mate precedes investment in meiosis, providing greater certainty of copulation and increasing the odds of the successful production of a viable zygote.

The novel combination of traits in raphid diatoms probably makes it easier to search for and find a genetically compatible mate. The diplontic life cycle of diatoms is characterized by long periods of asexual reproduction and short, periodic bouts of sexual reproduction. If vegetative cells cannot move, repeated vegetative divisions might lead to clonal patches of sibling cells, especially in benthic, nonmotile species that often live attached to substrates. This, in turn, might increase the chance of selfing or delay sexual reproduction until a nonclonal partner is encountered. The frequency of sexual reproduction and the rates of selfing or outcrossing in natural populations of diatoms are unknown and difficult to estimate. However, in culture, the majority of surveyed pennate diatoms are heterothallic, with mating occurring only between genetically compatible clones (Chepurnov & Mann, 2004; Chepurnov *et al*., 2004; but see, e.g., Davidovich *et al*., 2010, 2017). This led to the hypothesis that heterothally is the ancestral condition in pennates (Chepurnov *et al*., 2004). Moreover, when selfing has been observed and the progeny followed for a few generations thereafter, the cultured strains experienced inbreeding depression, resulting in reduced rates of gamete fusion and inviable zygotes (e.g. Chepurnov & Mann, 1999). It follows that selfing is probably rare in natural populations, and the various motility mechanisms that have evolved in different life history stages (gametes vs vegetative cells) might represent alternative strategies for increasing the frequency or efficiency of sex and outcrossing. The combination of life history and motility traits present in raphid pennate diatoms suggests that sexual reproduction is both more frequent and more efficient in this lineage compared with clades with nonmotile vegetative cells.

This interaction between locomotion and life history had lasting consequences for the ways in which raphid diatoms interact with conspecifics, heterospecifics and the environment. Raphe‐enabled motility relies on an actin–myosin system (Poulsen *et al*., 1999), and is directional, reversible and much faster than other forms of movement present in some nonraphid diatoms (Pickett‐Heaps *et al*., 1986, 1991; Kooistra *et al*., 2003). Active motility in vegetative cells enabled directed movement towards microhabitats with specific light, nutrient and temperature conditions (Cohn *et al*., 2015; Bondoc *et al*., 2016a), diurnal and tidal migrations (Palmer & Round, 1967), gravitactic behaviors (Frankenbach *et al*., 2014), predator avoidance (Kingston, 1999) and pheromonal migration (Sato *et al*., 2011; Gillard *et al*., 2013; Bondoc *et al*., 2016b; Moeys *et al*., 2016; Basu *et al*., 2017). This broad range of ecological benefits – unavailable to nonraphid diatoms – expanded the repertoire of habitats available for colonization, creating new opportunities for niche specialization. Active motility, and its tight association with substrate, might have lowered the overall rates of passive dispersal, reducing long‐range connectivity between populations, ultimately leading to greater isolation between local populations. Finally, the potentially higher frequency of sexual reproduction might have contributed to faster rates of adaptive divergence and the maintenance of reproductive isolation (Barraclough *et al*., 2003). Overall, the ability to move in response to both biotic and environmental stimuli appears to have provided greater potential for adaptive change and improved flexibility in dealing with habitat complexity in benthic habitats, where raphid diatoms thrive. Ultimately, the benefits of this novel locomotory trait might have contributed to the elevated diversification rate found in raphid pennate diatoms (Figs 2, 3, 4).

Although the myriad benefits of raphe‐enabled motility have been thoroughly characterized (Consalvey *et al*., 2004), these associations are nevertheless correlative and with a sample size of one (i.e. the gain of a raphe occurred just once in diatom evolution). This highlights a long‐standing challenge in comparative evolutionary biology. Namely, it is impossible to derive statistical support for an association between a trait and property, whether it is diversification or the evolution of another trait, when the focal trait evolved once (Maddison & FitzJohn, 2015; Beaulieu & O'Meara, 2016). Moreover, although it is likely that locomotion had a large effect on diatom evolution, diversification within raphid pennate diatoms was certainly influenced by other environmental, ecological and genetic factors. In this regard, the diversification of raphe‐bearing pennate diatoms resembles that of many other exceptionally diverse clades (e.g. flowering plants and insects), whose evolutionary success appears to be linked, at least superficially, to innovations that evolved only once. A more complete understanding of the diversification of diatoms, including raphid pennates, will need to account for the synergistic effects of other traits, biogeography and environmental changes, whose combined influence probably contributed to their diversification (Donoghue & Sanderson, 2015).

## Author contributions

T.N., J.M.B. and A.J.A. designed the study. T.N. collected the data and performed the analyses. T.N., J.M.B. and A.J.A. wrote the manuscript.

## Supporting information

Please note: Wiley Blackwell are not responsible for the content or functionality of any Supporting Information supplied by the authors. Any queries (other than missing material) should be directed to the *New Phytologist* Central Office.

**Fig. S1** Phylogeny of 1151 diatoms and 20 outgroups reconstructed using an 11‐gene dataset.
**Fig. S2** Diatom diversification estimated from first–last occurrence data for Cenozoic fossils.
**Fig. S3** Granularity of fossil data and its effect on the phylogenetic estimates of diversification.
**Fig. S4** Diatom diversification at predefined ranks with diversity data from AlgaeBase.
**Fig. S5** Discrete shifts and temporal trends of diversification across diatoms with diversity data from AlgaeBase.
**Table S1** Sequence data for diatoms and Parmales
**Table S2** Properties of the phylogenetic dataset, partitioning and model selection
**Table S3** ;Minimum and maximum bounds for the calibration of internal nodes
