# Supplementary material for: Accelerated diversification is related to life history and locomotion in a hyperdiverse lineage of microbial eukaryotes (Diatoms, Bacillariophyta)
Source: New Phytol. 2018 Apr 6;219(1):462–73. doi: 10.1111/nph.15137 (PMC6099383; doi:10.1111/nph.15137)
Supplement: Supplementary file 1 — Fig. S1 Phylogeny of 1151 diatoms and 20 outgroups reconstructed using an 11‐gene dataset. Fig. S2 Diatom diversification estimated from first–last occurrence data for Cenozoic fossils. Fig. S3 Granularity of fossil data and its effect on the phylogenetic estimates of diversification. Fig. S4 Diatom diversification at predefined ranks with diversity data from AlgaeBase. Fig. S5 Discrete shifts and temporal trends of diversification across diatoms with diversity data from AlgaeBase. Table S1 Sequence data for diatoms and Parmales Table S2 Properties of the phylogenetic dataset, partitioning and model selection Table S3 ;Minimum and maximum bounds for the calibration of internal nodes [file NPH-219-462-s001.pdf]

New Phytologist **Supporting Information**

Article title: Accelerated diversification is related to life history and locomotion in a hyperdiverse lineage of microbial eukaryotes (Diatoms, Bacillariophyta)

Authors: Teofil Nakov, Jeremy M. Beaulieu, Andrew J. Alverson

The following Supporting Information is available for this article:

Fig. S1. Phylogeny of 1,151 diatoms and 20 outgroups reconstructed using an 11-gene dataset.

Fig. S2. Diatom diversification estimated from first-last occurrence data for Cenozoic fossils.

Fig. S3. Granularity of fossil data and its effect phylogenetic estimates of diversification.

Fig. S4. Diatom diversification at predefined ranks with diversity data from AlgaeBase.

Fig. S5. Discrete shifts and temporal trends of diversification with diversity data from AlgaeBase.

Table S1 Sequence data for diatoms and Parmales.

Table S2 Properties of the phylogenetic dataset, partitioning, and model selection.

Table S3 Minimum and maximum bounds for calibration of internal nodes.

Fig. S1 Phylogeny of diatoms reconstructed using an 11-gene dataset for 1,151 diatoms and 20 outgroup heterokont taxa. Shown is the highest-scoring maximum likelihood phylogeny from 100 optimizations. Numbers at internal nodes are bootstrap support as estimated with IQtree's ultrafast bootstrap method (1000 pseudoreplicates).

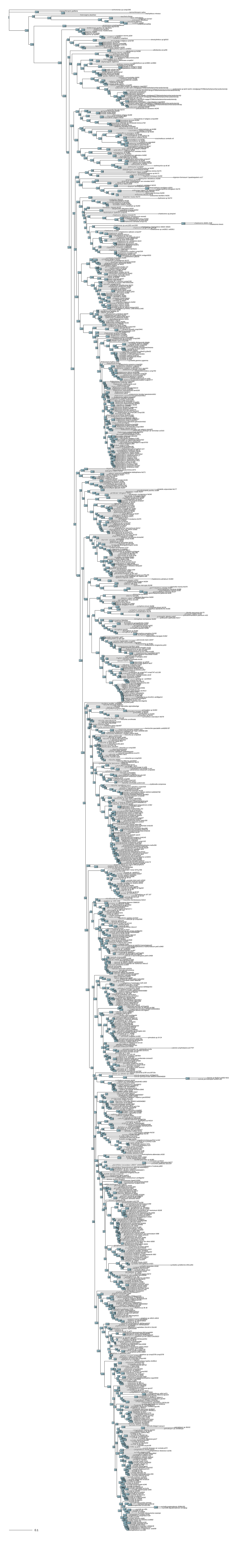

Fig. S2 Diatom diversification estimated from first-last occurrence data for Cenozoic fossils from the Barron Diatom Catalog. The data were binned into discrete, non-overlapping intervals with durations of 0.5, 1, 2.5, and 5.0 million years. Shown are medians calculated over the entire time span of the fossil data for each bin size.

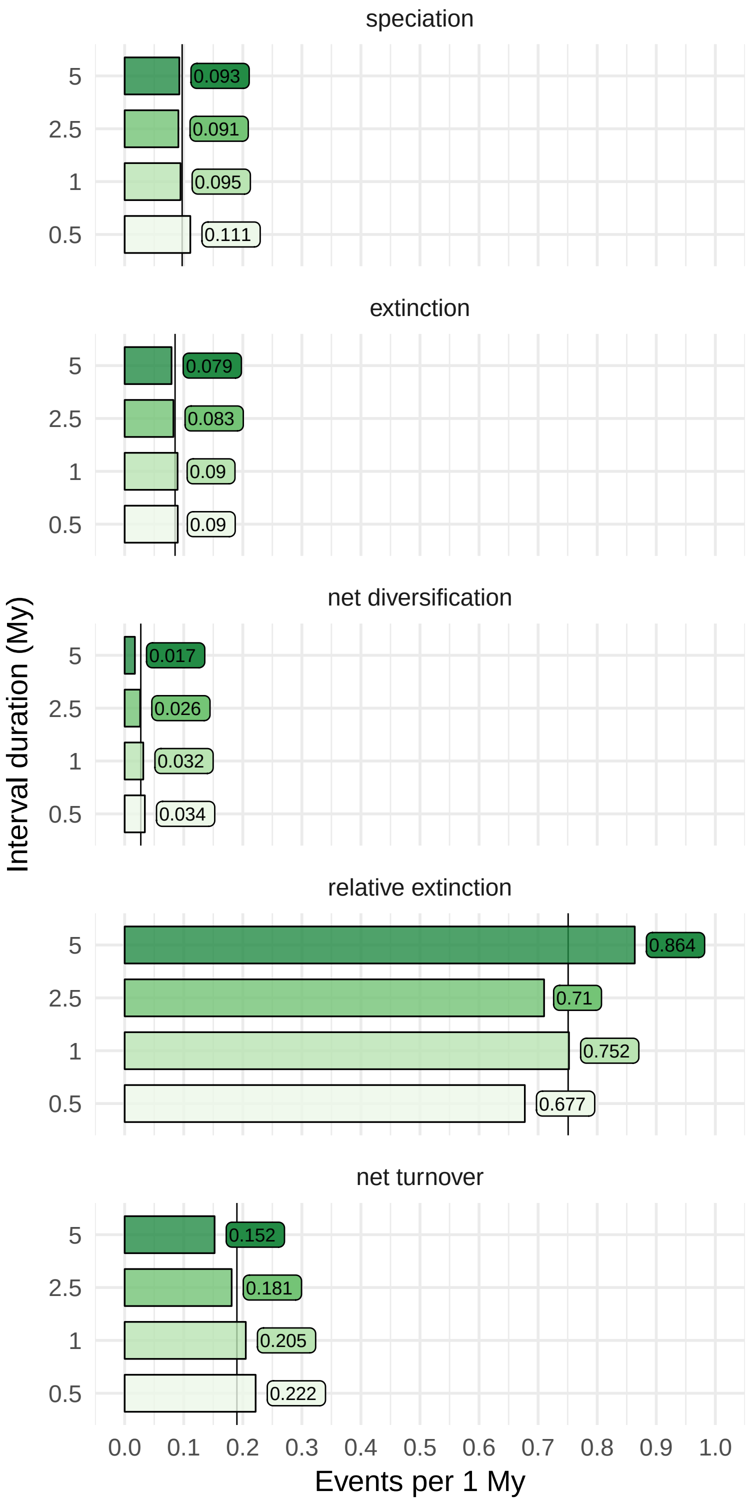
speciation
0.093
0.091
0.095
0.111
extinction
0.079
0.083
0.09
0.09
net diversification
0.017
0.026
0.032
0.034
relative extinction
0.864
0.71
0.752
0.677
net turnover
0.152
0.181
0.205
0.222
5
2.5
1
0.5
Interval duration (My)
0.0
0.1
0.2
0.3
0.4
0.5
0.6
0.7
0.8
0.9
1.0
Events per 1 My

Fig. S3 Granularity of fossil data and its effect phylogenetic estimates of diversification. Net diversification rate for diatom clades estimated using fossil relative extinction, crown or stem age, and standing diversity. Fossil relative extinction was estimated from first-last occurrence data were binned into 0.5, 1.0, 2.5, and 5.0 million year intervals. Extant species number data were from DiatomBase.

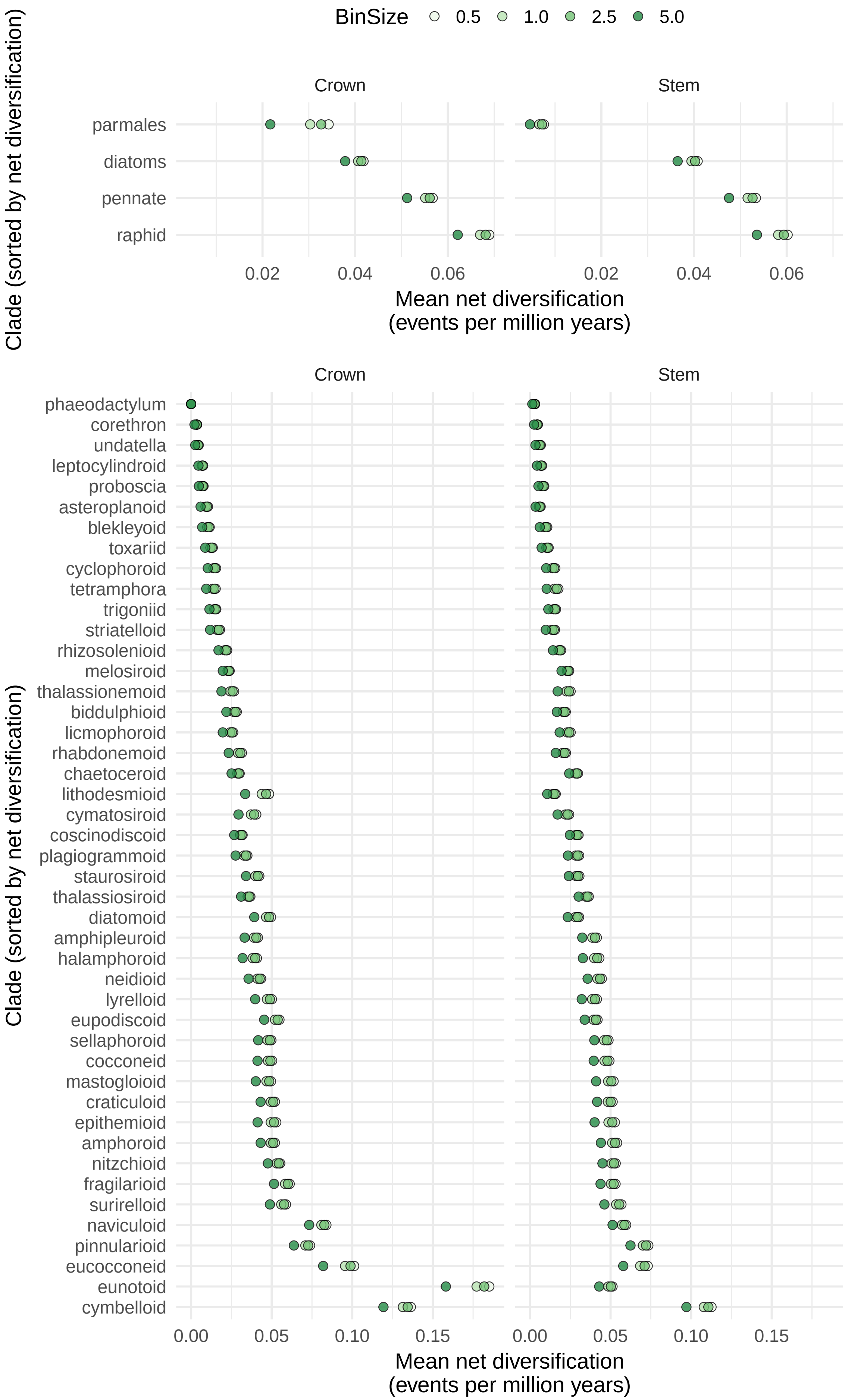
BinSize
0.5
1.0
2.5
5.0
Crown
Stem
parmales
diatoms
pennate
raphid
0.02
0.04
0.06
Mean net diversification
(events per million years)
Clade (sorted by net diversification)
phaeodactylum
corethron
undatella
leptocylindroid
proboscia
asteroplanoid
blekleyoid
toxariid
cyclophoroid
tetramphora
trigoniid
striatelloid
rhizosolenioid
melosiroid
thalassionemoid
biddulphioid
licmophoroid
rhabdonemoid
chaetoceroid
lithodesmioid
cymatosiroid
coscinodiscoid
plagiogrammoid
staurosiroid
thalassiosiroid
diatomoid
amphipleuroid
halamphoroid
neidioid
lyrelloid
eupodiscoid
sellaphoroid
cocconeid
mastogloioid
craticuloid
epithemioid
amphoroid
nitzchioid
fragilarioid
surirelloid
naviculoid
pinnularioid
eucocconeid
eunotoid
cymbelloid
0.00
0.05
0.10
0.15

Fig. S4 Diatom diversification at predefined ranks with diversity data from AlgaeBase. Diatom diversification based on fossil and phylogenetic data at predefined clades. **a.** Rates of speciation (birth, *b*), extinction (death, *d*) and species turnover ($\tau = b+d$) based on first–last occurrence data for Cenozoic diatoms from the Barron Diatom Catalog. **b.** Confidence intervals (95%) for species richness of pre-defined diatom clades, based on crown ages, standing diversity, and a relative extinction ($\varepsilon=d/b$) of ε=0.751. Line types correspond to different approximations of total diatom diversity (see Methods). Species richness from AlgaeBase is plotted against crown age (million years ago, Mya) and color-coded by the combination of reproductive mode and locomotion. Error bars span the minimum and maximum crown ages of clades across bootstrap trees in million years (My). The clades of all diatoms (D), pennate diatoms (P), and raphid diatoms (R) are shown as open squares. Clades younger than 10 My or having fewer than five species are omitted. **c.** Net diversification rates ($r = b–d$), averaged over bootstrap phylogenies and pooled based on the most-inclusive monophyletic groups with a certain combination of reproductive and locomotory traits. Lines, boxes, and whiskers represent medians, inter-quartile range, and quartile values + 1.5 * quartile values, respectively. See labels in Fig. 1 for contents of oogamous nonmotile (ON), anisogamous nonmotile (AN), and anisogamous motile (AM) groups. Abbreviations: Oog = oogamous, Anisog = anisogamous, Nonmot = non-motile, Mot = Motile.

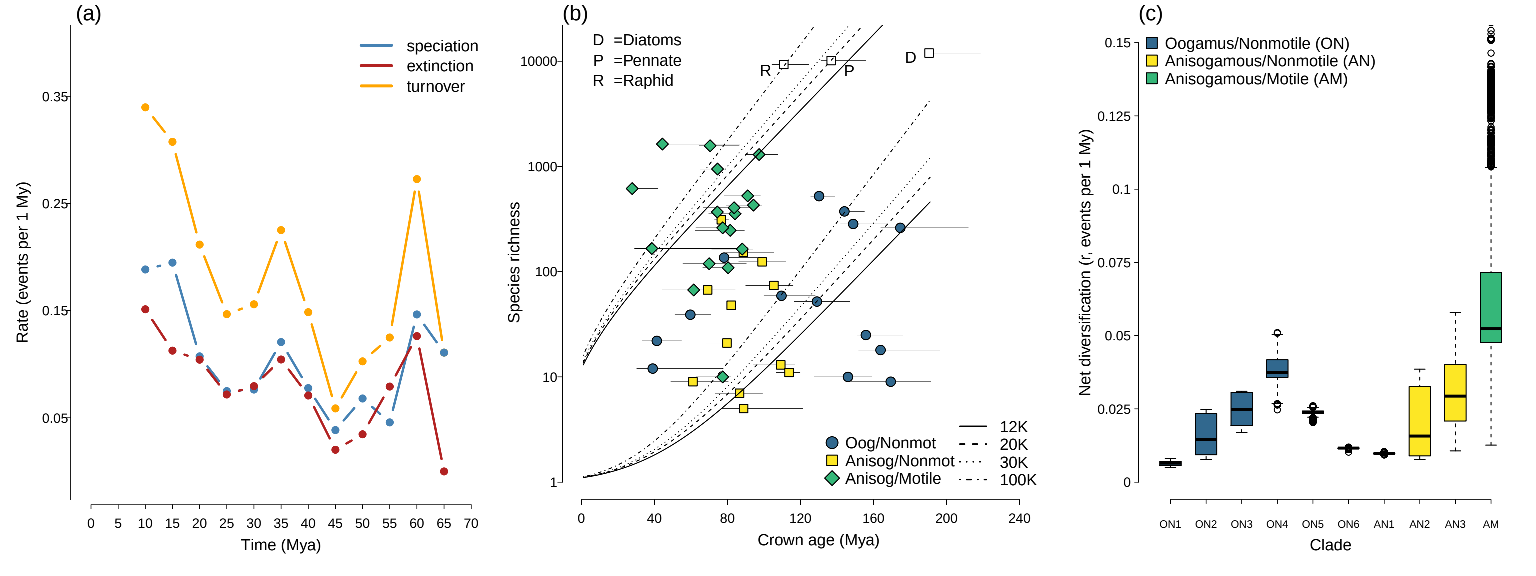
(a)
speciation
extinction
turnover
Rate (events per 1 My)
Time (Mya)
(b)
D =Diatoms
P =Pennate
R =Raphid
Species richness
Crown age (Mya)
Oog/Nonmot
Anisog/Nonmot
Anisog/Motile
12K
20K
30K
100K
(c)
Oogamus/Nonmotile (ON)
Anisogamous/Nonmotile (AN)
Anisogamous/Motile (AM)
Net diversification (r, events per 1 My)
Clade
ON1
ON2
ON3
ON4
ON5
ON6
AN1
AN2
AN3
AM

Fig. S5 Discrete shifts and temporal trends of diversification across diatoms with species richness data from AlgaeBase. **a**. MEDUSA reconstruction of rates of (birth – death, $r = b\text{-}d$, events per million years) across the genus-level phylogeny of diatoms+Parmales. Triangle size is proportional to the number of species per genus (from AlgaeBase), and the shading corresponds to the estimated net diversification rate. Rate shifts detected in ≥50% of trees are shown. **b-d**. Temporal trends in net diversification, relative extinction ($\varepsilon = d/b$), and turnover ($\tau = b+d$) as estimated by the genus-level MEDUSA analysis. Trend lines were calculated by slicing the bootstrap phylogenies into 1 million years (My) intervals and averaging the branch-associated parameters for each slice across trees. Trendlines are shown for the most inclusive clades with a combination of reproductive and locomotory traits and match the groups in Fig. 2c and clade labels in Fig. 1. Rates estimated from the Cenozoic marine fossil record (bin size 5 My) were smoothed using non-parametric local polynomial regression (LOESS) but are otherwise not different from Fig. 2a. The timing of the evolution of active motility (the range of crown age estimates for of raphid pennates across bootstrap phylogenies) is shown as a blue rectangle.

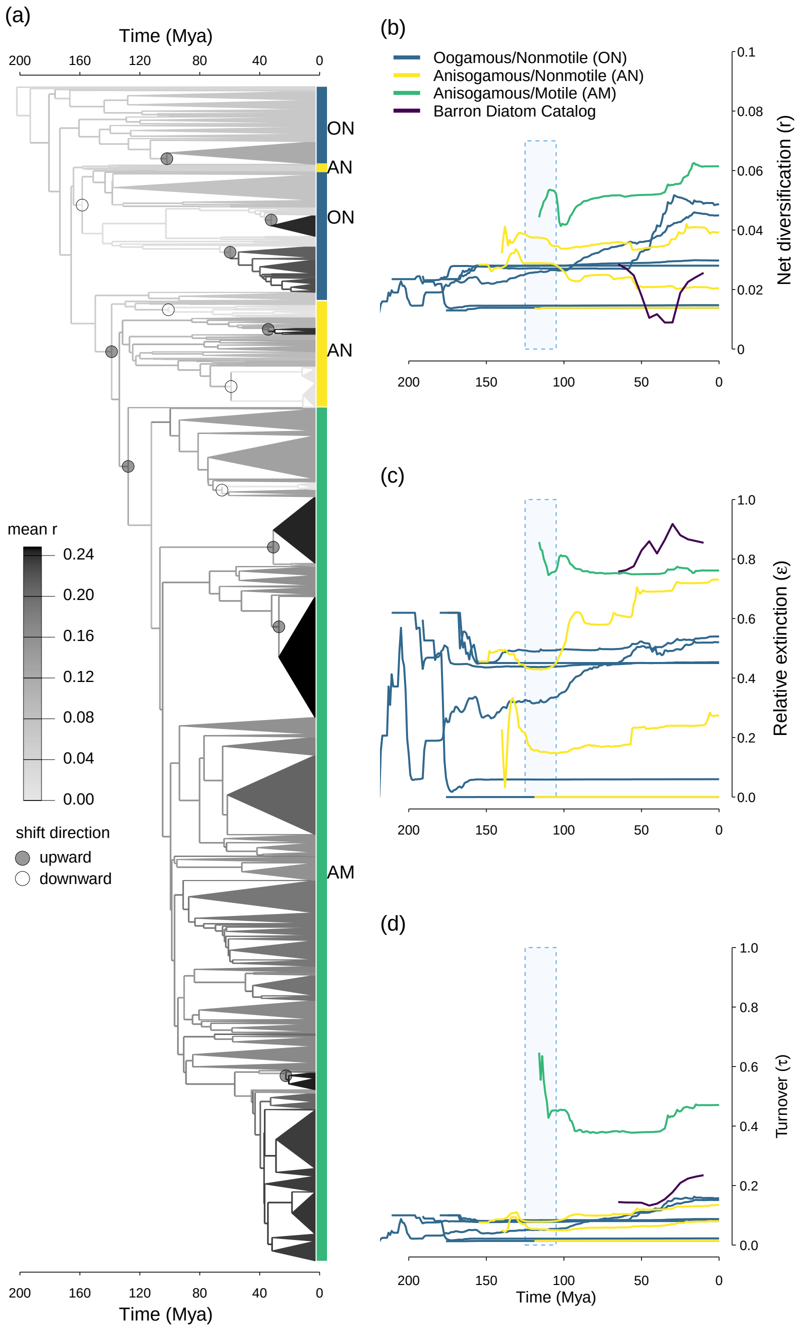

(a)
Time (Mya)
200
160
120
80
40
0
ON
AN
ON
AN
AM
mean r
0.24
0.20
0.16
0.12
0.08
0.04
0.00
shift direction
upward
downward
(b)
Oogamous/Nonmotile (ON)
Anisogamous/Nonmotile (AN)
Anisogamous/Motile (AM)
Barron Diatom Catalog
Net diversification (r)
0.1
0.08
0.06
0.04
0.02
0
200
150
100
50
0
(c)
Relative extinction (ε)
1.0
0.8
0.6
0.4
0.2
0.0
(d)
Turnover (τ)
Time (Mya)

Table S1 Sequence data for diatoms and Parmales as downloaded from the European Nucleotide Archive on May 20, 2016 and after quality control and redundancy removal.

Compartment: gene	Total sequences downloaded: diatoms+Parmales	Total sequences in final dataset	Total length of final alignment
chloroplast: *atpB*	207	183	1293
chloroplast: *psaA*	306	205	1584
chloroplast: *psaB*	266	230	1947
chloroplast: *psbA*	427	322	954
chloroplast: *psbC*	631	463	1239
chloroplast: *rbcL*	2761	850	1470
chloroplast: SSU rRNA (16s)	341	51	1413
mitochondrion: *cob*	147	82	711
mitochondrion: *cox1*	231	38	681
nucleus: LSU rRNA (28s)	863	163	371
nucleus: SSU rRNA (18s)	3294	995	1594

Table S2 Properties of the phylogenetic dataset, partitioning, and selection of models of nucleotide substitution. The best models were those with lowest Bayesian Information Criterion (BIC) scores as calculated by the model selection routine available in IQ-TREE.

Partition	#Seqs	#Sites	#Site patterns	Constant Sites	Best model
nuclear+plastid rRNA	1020	3378	2225	49%	TIM2+R9
plastid 1st codon	869	2829	1867	55%	GTR+R9
plastid 2nd codon	869	2829	1449	73%	TVM+R6
plastid 3rd codon	869	2829	2791	4%	GTR+R10
mitochondrial 1st codon	120	464	286	52%	GTR+I+G4
mitochondrial 2nd codon	120	464	188	76%	TVM+R4
mitochondrial 3rd codon	120	464	446	7%	TIM+R4

Table S3 Minimum and maximum bounds for calibration of internal nodes. The penultimate distance (PenD) is the time between the first (FA) and next appearance (NA) of a lineage in the fossil record (Norris *et al.*, 2015). The ghost lineage time (GLin) is the difference between the first appearances of two sister lineages (Norris *et al.*, 2015). The minimum and maximum bounds for the ages of calibrated nodes were taken as the 5% and 95% quantiles of a prior distribution derived from the observed PenD or GLin times (Methods). When information for calculating PenD or GLin was missing, we applied only a minimum bound (MIN). All times are in millions of years (My).

Bracket 1	Bracket 2	Type	Description	Min	Max
Bolidomonas	*Triparma*	PEND	FA(Parmales=66) - NA(Parmales=34); (Konno & Jordan, 2012)	66	155
Bolidomonas	*Campylodiscus*	GLIN	FA(diatoms=190) - FA(Parmales=66); (Rothpletz, 1896; Konno & Jordan, 2012)	190	350
Corethron	*Campylodiscus*	PEND	FA(diatoms=190) - NA(diatoms=135); (Rothpletz, 1896; Harwood *et al.*, 2007)	190	302
Melosira	*Aulacoseira*	GLIN	FA(*Melosira*=125) - FA(*Archepyrgis*=115); (Geroch, 1978; Gersonde & Harwood, 1990)	125	178
Stephanopyxis	*Paralia*	GLIN	FA(*Stephanopyxis*=100) - FA(*Hyalodiscus*=83.6); (Girard *et al.*, 2009; Witkowski *et al.*, 2011)	100	166
Aulacodiscus	*Stellarima*	GLIN	FA(*Aulacodiscus*=125) - FA(*Stellarima*=83.6); (Geroch, 1978; Witkowski *et al.*, 2011)	125	224
Rhizosolenia	*Aulacodiscus*	GLIN	FA(*Aulacodiscus*=125) - FA(*Rhizosolenia*=90); (Geroch, 1978; Damsté *et al.*, 2004)	125	217
Actinocyclus	*Actinoptychus*	GLIN	FA(*Actinoptychus*=70.6) – FA(*Actinocyclus*=56.5); (Lazarus, 1994)	70	132
Stellarima	*Coscinodiscus*	GLIN	FA(*Stellarima*=83.6) – FA(*Coscinodiscus*=77.05); (Lazarus, 1994)	83	128
Trieres	*Odontella*	GLIN	FA(*Triceratium*=77.05) – FA(*Trieres*=66); (Hajós & Stradner, 1975; Harwood, 1988)	77	133
Amphitetras	*Amphipentas*	MIN	Neptune FA of *Amphitetras*; (Lazarus, 1994)	9.16	
Hemiaulus	*Chaetoceros*	GLIN	FA(*Hemiaulus*) - FA(*Chaetoceros*=55); (Fenner, 1994; Witkowski *et al.*, 2011)	100	203
Eucampia	*Cerataulina*	MIN	Neptune FA of *Eucampia*; (Lazarus, 1994)	20.05	

Cymatosira	*Campylosira*	MIN	Neptune FA of *Cymatosira*; (Lazarus, 1994)	44.18	
Lithodesmium	*Bellerochea*	MIN	Neptune FA of *Lithodesmium*; (Lazarus, 1994)	29.96	
Porosira	*Stephanodiscus*	PEND	FA(*Praethalassiosirposis*=115) - NA(*Gladiopsis*=112); (Nikolaev *et al.*, 2001)	115	146
Porosira	*Lauderia*	MIN	Neptune FA of *Porosira*; (Lazarus, 1994)	9	
Thalassiosira	*Shionodiscus*	MIN	Neptune FA of *Shionodiscus*; (Lazarus, 1994)	14.6	
Stephanodiscus	*Cyclostephanos*	GLIN	FA(*Stephanodiscus*=16.36) – FA(*Cyclostephanos*=5); Neptune FA of Steph; (Alverson, 2014)	16	73
Bacterosira	*Detonula*	MIN	Neptune FA of *Bacterosira*; (Lazarus, 1994)	8.35	
Biddulphia	*Attheya*	MIN	Neptune FA of *Biddulphia*; (Lazarus, 1994)	70.6	
Striatella	*Campylodiscus*	MIN	FA of Surirella and Campylodiscus; (Hajós & Stradner, 1975; Sims *et al.*, 2006)	75	
Thalassiothrix	*Thalassionema*	MIN	Neptune FA of *Thalassiotrix* and *Thalassiosinema*; (Lazarus, 1994)	56.5	
Synedra	*Fragilaria*	GLIN	FA(*Synedra*=56.4) – FA(*Fragilaria*=37.7); (Lazarus, 1994)	56	126
Synedropsis	*Grammonema*	MIN	FA of *Synedropsis*; (Stickley *et al.*, 2009)	47	
Tabularia	*Catacombas*	MIN	Neptune FA of *Tabularia*; (Lazarus, 1994)	9.18	
Grammatophora	*Hyalosira*	PEND	FA(*Grammatophora*=40) – NA(*Grammatophora*=34.18); (Edwards, 1991)	40	82
Rhaphoneis	*Delphineis*	GLIN	FA(*Rhaphoneis*=56.4) – FA(*Delphineis*=37.7); (Lazarus, 1994)	38	53
Talaroneis	*Psammoneis*	MIN	Neptune FA of *Talaroneis*; (Lazarus, 1994)	20.73	
Opephora	*Staurosirella*	MIN	Neptune FA of *Opephora*; (Lazarus, 1994)	20.05	
Plagiogramma	*Dimeregramma*	GLIN	FA(*Dimeregramma*=21.8) – FA(*Plagiogramma*=14.6); (Lazarus, 1994)	21	68
Asteroplanus	*Asterionellopsis*	MIN	FA of *Asterionellopsis*; (Schrader & Gersonde, 1978)	5.3	
Undatella	*Campylodiscus*	MIN	FA of raphid pennate diatom; (Singh *et al.*, 2006)	66	
Bacillaria	*Denticula*	MIN	FA of several genera at 56.5 in Neptune dataset; (Lazarus, 1994)	56.47	
Navicula	*Pleurosigma*	GLIN	FA(*Navicula*=44.36) – FA(*Pleurosigma*=37.97); (Lazarus, 1994)	44	88
Lyrella	*Cocconeis*	GLIN	FA(*Cocconeis*=30.33) – FA(*Lyrella*=28.28); (Lazarus, 1994)	30	57
Gomphonema	*Encyonema*	GLIN	FA(*Encyonema*=14.18) – FA(*Gomphonema*=13.09); (Lazarus, 1994)	14	34
Coronia	*Campylodiscus*	MIN	FA of *Campylodiscus*; (Reinhold, 1937; Hajós & Stradner, 1975)	16	

References

Alverson AJ. **2014**. Timing marine–freshwater transitions in the diatom order Thalassiosirales. *Paleobiology* **40**: 91–101.

Damsté JSS, Muyzer G, Abbas B, Rampen SW, Massé G, Allard WG, Belt ST, Robert J-M, Rowland SJ, Moldowan JM, *et al.* **2004**. The rise of the rhizosolenid diatoms. *Science* **304**: 584–587.

Edwards AR. **1991**. *The Oamaru Diatomite*. New Zealand Geological Survery paleontological bulletin, 64. DSIR Geology & Geophysics. Lower Hutt, New Zealand.

Fenner J. **1994**. Diatoms of the Fur Formation, their taxonomy and biostratigraphic interpretation–results from the Harre borehole, Denmark. *Aarhus Geoscience* **1**: e131.

Geroch S. **1978**. Lower Cretaceous diatoms in the Polish Carpathians. *Annales Societatis Geologorum Poloniae* **48:** 283-295.

Gersonde R, Harwood DM. **1990**. 25. Lower Cretaceous diatoms from ODP LEG 113 Site 693 (Weddel sea). Part 1: Vegetative cells. In: Barker, P. F., Kenneth, J. P. et al. *Proceedings of the Ocean Drilling Program, Scientific Results* **113:** 365–402.

Girard V, Saint Martin S, Saint Martin J-P, Schmidt AR, Struwe S, Perrichot V, Breton G, Néraudeau D. **2009**. Exceptional preservation of marine diatoms in upper Albian amber. *Geology* **37**: 83–86.

Hajós M, Stradner H. **1975**. Late Cretaceous Archaeomonadaceae, Diatomaceae, and Silicoflagellatae from the South Pacific Ocean, Deep Sea Drilling Project, Leg 29, Site 275. *Initial Reports of the Deep Sea Drilling Project* **29**: 913–1009.

Harwood DM. **1988**. Upper Cretaceous and lower Paleocene diatom and silicoflagellate biostratigraphy of Seymour Island, eastern Antarctic Peninsula. *Geological Society of America*

Memoirs **169**: 55–130.

Harwood DM, Nikolaev VA, Winter DM. **2007**. Cretaceous Records of Diatom Evolution, Radiation, and Expansion. *The Paleontological Society Papers* **13**: 33–59.

Konno S, Jordan RW. **2012**. Parmales. In: John Wiley & Sons, Ltd, ed. eLS. Chichester, UK: John Wiley & Sons, Ltd.

Lazarus D. **1994**. Neptune: A marine micropaleontology database. *Mathematical geology* **26**: 817–832.

Nikolaev VA, Kociolek JP, Fourtanier E, Barron JA, Harwood DM. **2001**. Late Cretaceous diatoms (Bacillariophyceae) from the Marca shale member of the Moreno formation, California. *Occasional papers of the California Academy of Sciences* **152**: 1–119.

Norris RW, Strope CL, McCandlish DM, Stoltzfus A. **2015**. Bayesian priors for tree calibration: Evaluating two new approaches based on fossil intervals. *bioRxiv*: 014340.

Reinhold T. **1937**. Fossil diatoms of the Neogene of Java and their zonal distribution. *Verhandel. Geol. -Mijinbow. Geol. Ser.* **12**: 43–133.

Rothpletz A. **1896**. Ueber die Flysch-Fucoiden und einige andere fossile Algen, sowie über liasische, Diatomeen führende Hornschwämme. *Zeitschrift der Deutschen Geologischen Gesellschaft* **48**: 854–914.

Schrader H, Gersonde R. **1978**. The late Messinian Mediterranean brackish to freshwater environment diatom floral evidence. *In Zachariasse et al. Microplaeontological counting methods and In Hsü, K. , Montadert, L., et al. Init. Repts DSDPPt. 1): Washington (US Govt. Printing Office)* **42**: 761–775.

Sims PA, Mann DG, Medlin LK. **2006**. Evolution of the diatoms: Insights from fossil, biological and molecular data. *Phycologia* **45**: 361–402.

Singh RS, Stoermer EF, Kar R. **2006**. Earliest freshwater diatoms from the Deccan Intertrappean (Maastrichtian) sediments of India. *Micropaleontology* **52**: 545–551.

Stickley CE, St John K, Koç N, Jordan RW, Passchier S, Pearce RB, Kearns LE. **2009**. Evidence for middle Eocene Arctic sea ice from diatoms and ice-rafted debris. *Nature* **460**: 376–379.

Witkowski J, Harwood DM, Chin K. **2011**. Taxonomic composition, paleoecology and biostratigraphy of late Cretaceous diatoms from Devon Island, Nunavut, Canadian High Arctic. *Cretaceous Research* **32**: 277–300.
